# Adult Small Gut Intussusception Caused by *Ascaris* as a Lead Point

**DOI:** 10.4269/ajtmh.14-0351

**Published:** 2015-08-05

**Authors:** Shruti Thakur, Anupam Jhobta, Charu S. Thakur

**Affiliations:** Indira Gandhi Medical College and Hospital, Radiodiagnosis, Shimla, Himachal Pradesh, India

A 35-year-old woman presented with generalized abdominal pain for the past 2 years. The pain was intermittent and moderate in intensity. There was no anorexia, vomiting, or fever. Her bladder and bowel habits as well as physical and abdominal examination were normal.

The routine blood and urine tests were also unremarkable. The ultrasound done as a first-line imaging investigation showed normal solid organs with normal caliber of gut loops. However, contrast-enhanced computed tomography (CECT) was done to ascertain any pathology missed on sonography. CECT showed small gut intussusception with worms as lead point (Figure 1

**Figure 1.**
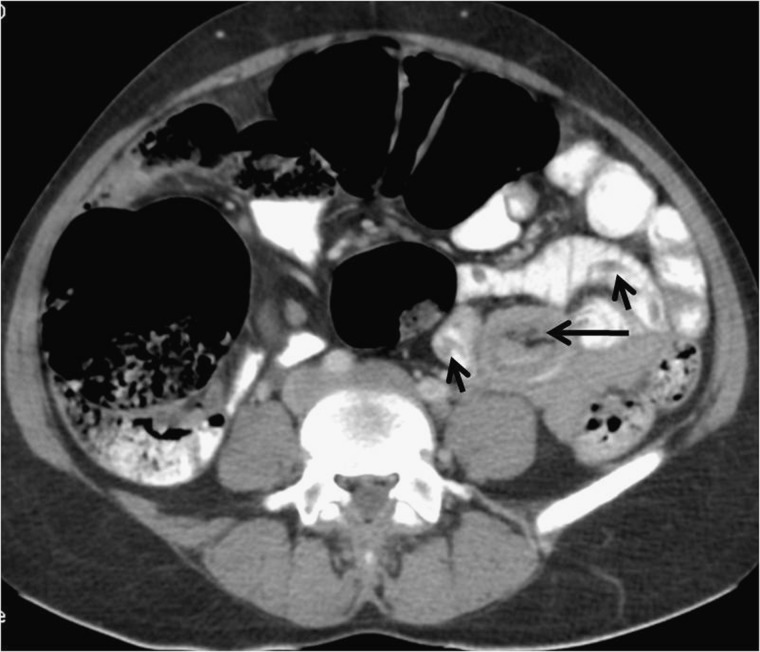

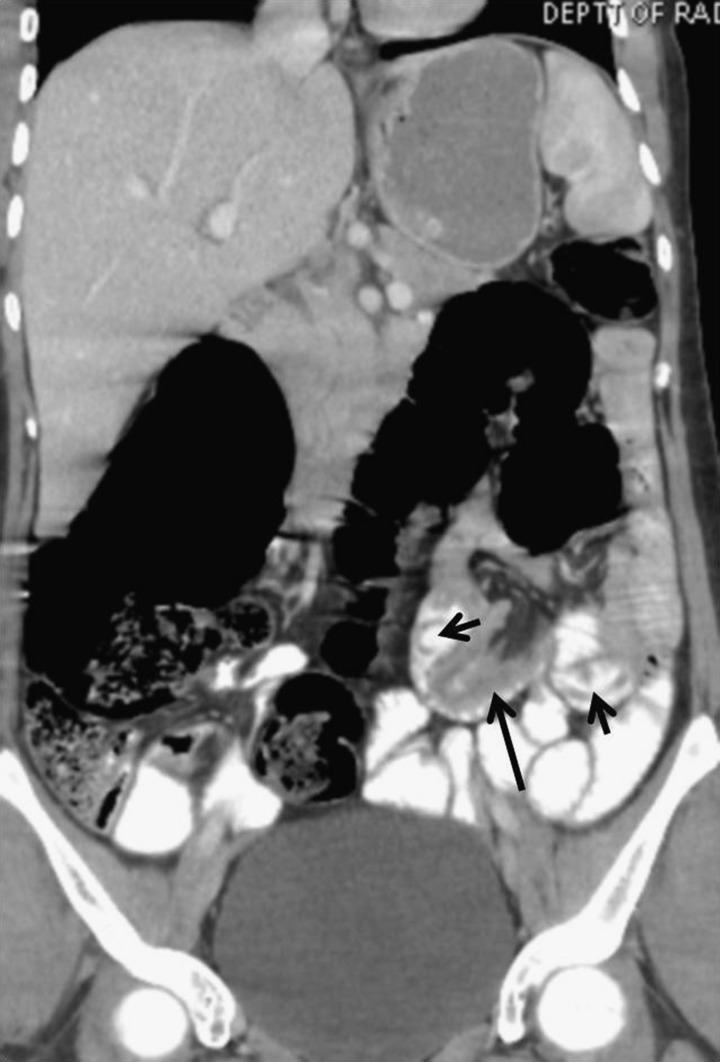
(**A**, **B**) CECT images show a part of small gut loop along with its mesentery invaginating into the lumen of contiguous gut loop giving “bowel-within-bowel” configuration suggestive of entero-enteric intussusception (long black arrow). No mass lesion is seen at the lead point of intussusception. There is no sign of bowel obstruction. A few tubular hypodense structures within the orally opacified gut are seen at the site of intussusception that look like worms (small black arrows).

). The patient was reviewed on ultrasound the next day by a consultant radiologist. The patient was prepared by making her drink 1.5 L water in 1 hour to distend the small gut by water as air hinders sonographic evaluation. Sonography showed no feature of intussusception that was seen on CECT, but the worms could be delineated (Figure 2

**Figure 2.**
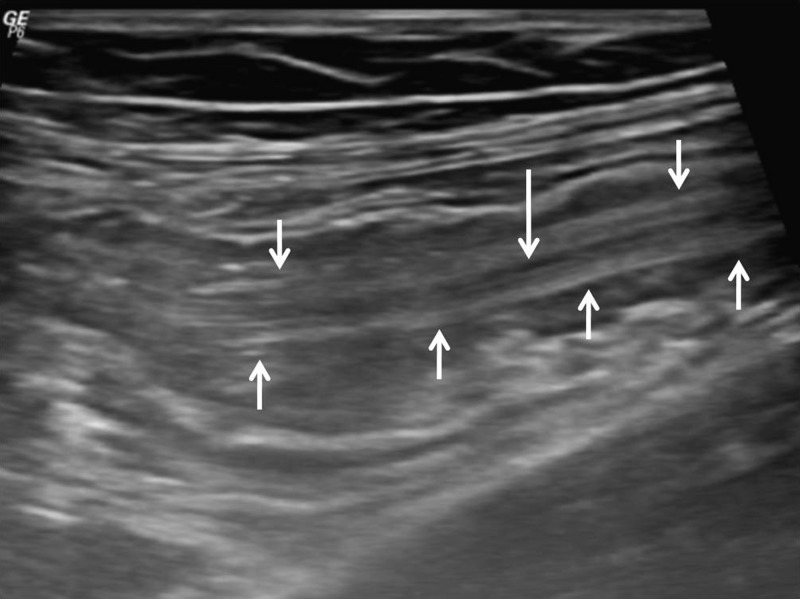

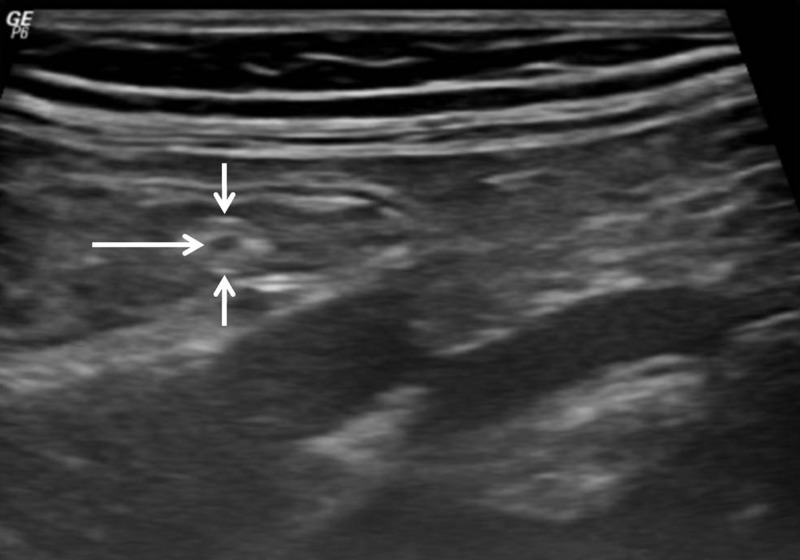
(**A**, **B**) Sonographic images show linear parallel echogenic structures that look like “railway tract” on longitudinal imaging and “bull's eye” on transverse imaging (small white arrows). There is no posterior acoustic shadowing. These tubular structures showed active movement on real time sonography, thereby confirming worm infestation. The central anechoic linear area represents the digestive tract of the worm (long white arrow).

). So it was a rare and interesting case of transient entero-enteric intussusception with worms as lead point. The patient's stool examination was positive for ova of *Ascaris lumbricoides*. There was no acute symptom so she was managed conservatively with oral albendazole.

*A. lumbricoides* is a common cause of bowel obstruction in children in tropics and subtropics (Supplemental Video). But *A. lumbricoides* as a cause of intussusception in adults is very unusual and described as a handful of case reports.^1^ The abdominal complications can be broadly categorized into intestinal and extraintestinal. The gastrointestinal complications include mechanical bowel obstruction, volvulus, intussusception, appendicitis, peritonitis, or even perforation.^2^ The extraintestinal abdominal complications are biliary colic, cholelithiasis, cholecystitis, liver abscess, and pancreatitis.

## Supplementary Material

Supplemental Video.
